# Biomimetic cell-derived nanocarriers in cancer research

**DOI:** 10.1186/s12951-022-01748-4

**Published:** 2022-12-22

**Authors:** Enrica Soprano, Ester Polo, Beatriz Pelaz, Pablo del Pino

**Affiliations:** Centro Singular de Investigación en Química Biolóxica e Materiais Moleculares (CiQUS), Universidade de Santiago de Compostela, 15705 Santiago de Compostela, Spain

**Keywords:** Biomimetic nanocarrier, Drug delivery, Intracellular delivery, Cancer therapy, Cell-membrane coating Nanoparticles

## Abstract

Nanoparticles have now long demonstrated capabilities that make them attractive to use in biology and medicine. Some of them, such as lipid nanoparticles (SARS-CoV-2 vaccines) or metallic nanoparticles (contrast agents) are already approved for their use in the clinic. However, considering the constantly growing body of different formulations and the huge research around nanomaterials the number of candidates reaching clinical trials or being commercialized is minimal. The reasons behind being related to the “synthetic” and “foreign” character of their surface. Typically, nanomaterials aiming to develop a function or deliver a cargo locally, fail by showing strong off-target accumulation and generation of adverse responses, which is connected to their strong recognition by immune phagocytes primarily. Therefore, rendering in negligible numbers of nanoparticles developing their intended function. While a wide range of coatings has been applied to avoid certain interactions with the surrounding milieu, the issues remained. Taking advantage of the natural cell membranes, in an approach that resembles a cell transfer, the use of cell-derived surfaces has risen as an alternative to artificial coatings or encapsulation methods. Biomimetic technologies are based on the use of isolated natural components to provide autologous properties to the nanoparticle or cargo being encapsulated, thus, improving their therapeutic behavior. The main goal is to replicate the (bio)-physical properties and functionalities of the source cell and tissue, not only providing a stealthy character to the core but also taking advantage of homotypic properties, that could prove relevant for targeted strategies. Such biomimetic formulations have the potential to overcome the main issues of approaches to provide specific features and identities synthetically. In this review, we provide insight into the challenges of nano-biointerfaces for drug delivery; and the main applications of biomimetic materials derived from specific cell types, focusing on the unique strengths of the fabrication of novel nanotherapeutics in cancer therapy.

**Graphical Abstract:**

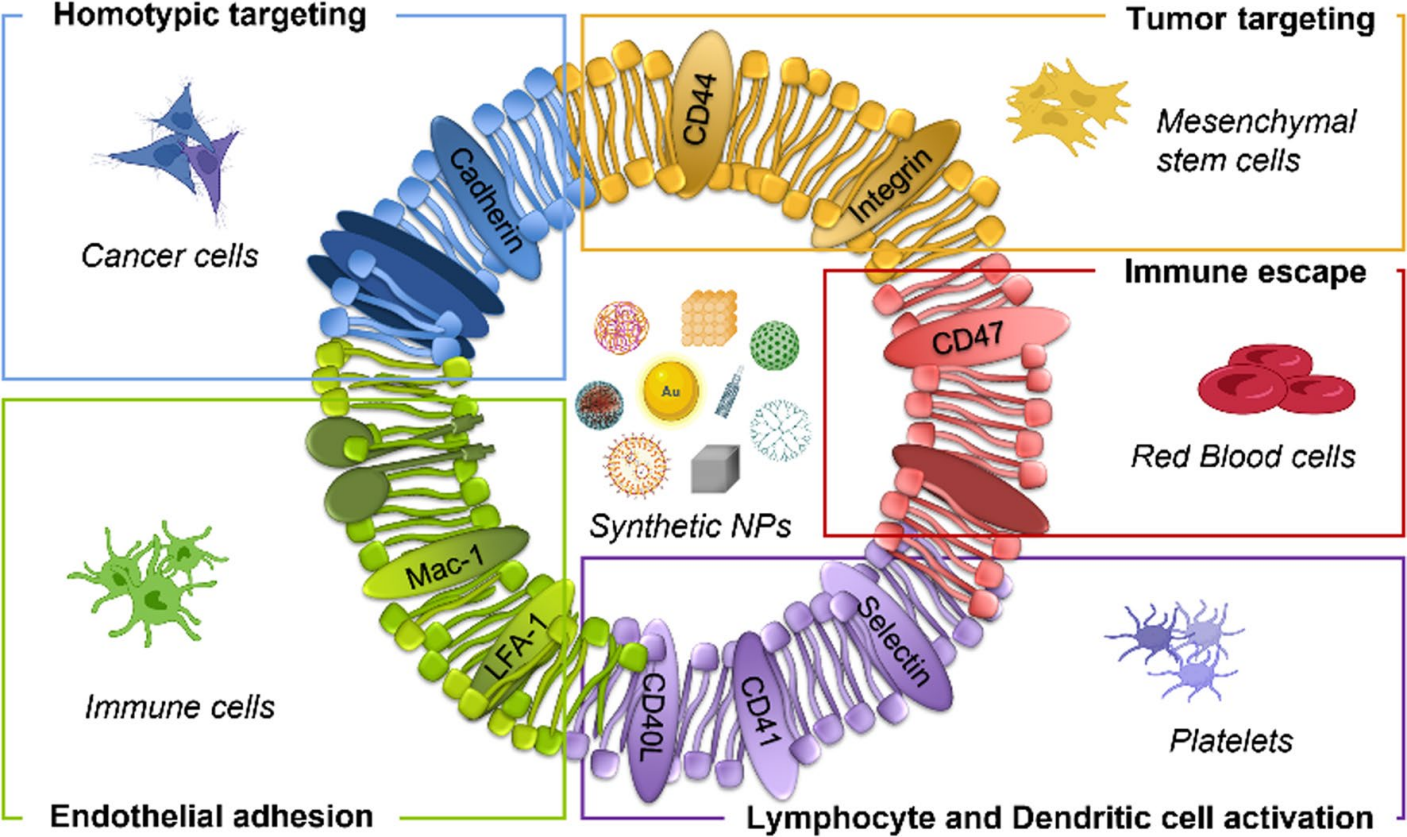

## Introduction

**Fig. 1:**
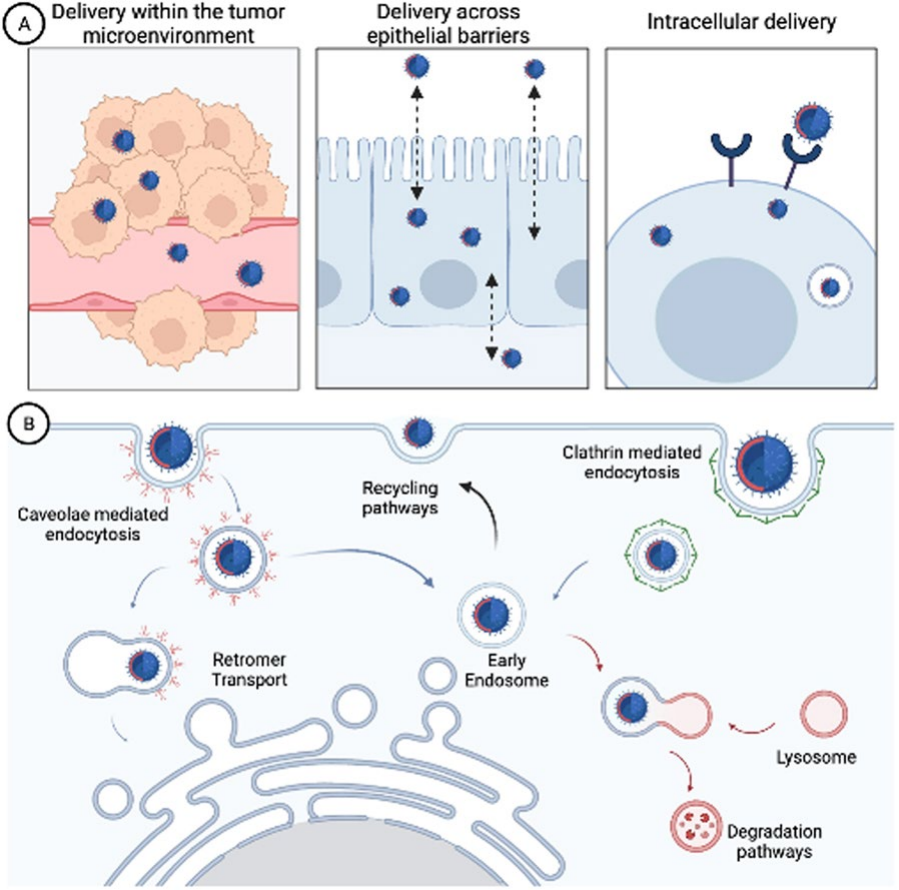
**A** Heterogeneous biological barriers that nanomaterials must overcome to successfully deliver drugs at precise locations: (1) tumor microenvironment, (2) crossing epithelial barriers and (3) cell targeting and intracellular delivery. **B **Representation of the main endocytic pathways

In the context of biomedical applications, nanobiotechnology allows the manipulation of materials at molecular levels, which aims to produce non-toxic bioactive nanodevices that have specificity toward a desired tissue and location. The advantages of using nanomaterials as drug delivery systems (DDS) are related to their small size, which allows them to cross biological barriers and small capillaries thus reaching targets of interest such as tissues, tumors, or individual cells (Fig. 1A) [1]. Furthermore, their modifiable structure offers the possibility of encapsulating the drug or conjugating it on the surface, by adsorption or chemical bond, thus protecting it from premature degradation and/or elimination in vivo and, at the same time, guaranteeing its solubility in the biological environment [2]. The dimensions of the nanosystems allow for a high surface area compared to their bulk materials counterparts, which improves the ability to bind to the surface molecules that attribute specific functionality to nanoparticles (NPs) [3]. This feature offers a great advantage to reach biological targets thanks to the conjugation with specific ligands (antibodies, peptides, etc.) [2]. In addition, the use of NPs as drug delivery vectors favors the accumulation of the drug in the site of therapeutic interest and reduces its dispersion in the body. Consequently, it allows not only decreased dosage frequency but also to reduce side effects, favoring patient compliance [4].

Based on the mechanisms through which the diseased tissue is reached, the DDS are broadly classified into passive and active targeting [5] Passive targeting is driven simply by the size and shape of the NPs, which determine their biodistribution and accumulation at the tissue level [6]. For this reason, passive targeting is mainly used to treat pathologies that alter the characteristics of the body’s tissues, as in the case of tumors [7, 8]. Passive permeation at the tumor tissue level is defined as the Enhanced Permeability and Retention effect (EPR). Because passive targeting does not rely on biochemical identification, it has the disadvantage of having low target specificity. On the other hand, active targeting involves engineering NP surface with specific molecules such as peptides, proteins, or antibodies to identify and bind to cell-specific ligands that are expressed on the cell membrane [5, 9]. To be successful, active targeting requires that the receptor of interest be exclusive of, or overexpressed by, the cells of the target tissues [10], to achieve a preferential drug accumulation in the diseased tissue and, thus, a selective therapeutic system. Targeting moieties such as antibodies [11], aptamers [12], transferrin [13], epidermal growth factor **(**EGF) [14], and folic acid [15], among others, are employed as ligands to promote the specific recognition of the cellular plasma membrane components. Targets can also be part of the intracellular components such as mitochondria [16], nuclei [17], or lysosomes [18].

Despite all the above-mentioned advantages, still many challenges that have limited the widespread success of conventional NP-based drug delivery systems (such as inflammation, off-target, and clearance) need to be faced. Therefore, research efforts remain aiming at engineering drug nanocarrier biointerfaces that provide the desired features to cross heterogeneous biological barriers and reach the target therapeutic sites. Biomimetic nanocarriers have the potential to improve circulation times, to transport across membranes, to improve solubility and stability of encapsulated cargos. These delivery nanoplatforms offer smart designs with sophisticated and organized self-assembled architectures with functional diversities and integrated stability [19]. Employing naturally derived nanocarriers such as exosomes, virus-like particles, or cell membrane-derived coatings has become a powerful innovative strategy capable of recreating complex architecture and cellular functionalities to overcome limitations of lipid-based, polymeric, and inorganic NPs [20, 21]. Improvements in designing smart delivery platforms could lead to develop efficient cancer nano-based therapies. In this review we discuss the main challenges and limitations of nano-based drug delivery platforms. We also summarize different cell-derived nanosystems to overcome these obstacles. Additionally, we discuss opportunities and challenges associated with the application and translation of cell-derived nanocarriers.

### Intracellular cargo delivery

In addition to the general in vivo improvements in terms of avoided sequestration by the reticuloendothelial system (RES), prolonged circulation time, and specific cell or tissue targeting, nanomaterials' internalization pathways must be taken into account, as intracellular delivery that require endosomal escape is a fundamental prerequisite for obtaining successful DDS [22]. In most cases, NPs are taken up through the processes of endocytosis. Depending on the entry pathway, endosomes can be recycled, and transported to extracellular space or to organelles such as lysosomes, Golgi apparatus, or mitochondria (Fig. 1B). In most cases, nanomaterials get trapped inside the endosomes and undergo protease-mediated degradation and exocytosis, resulting in a very limited fraction of successfully delivered molecules achieving their cytosolic target [23–25]. At this stage, specific and efficient methods of intracellular drug delivery are required, which represents one of the most relevant challenges for protein-based therapies.

**Fig. 2:**
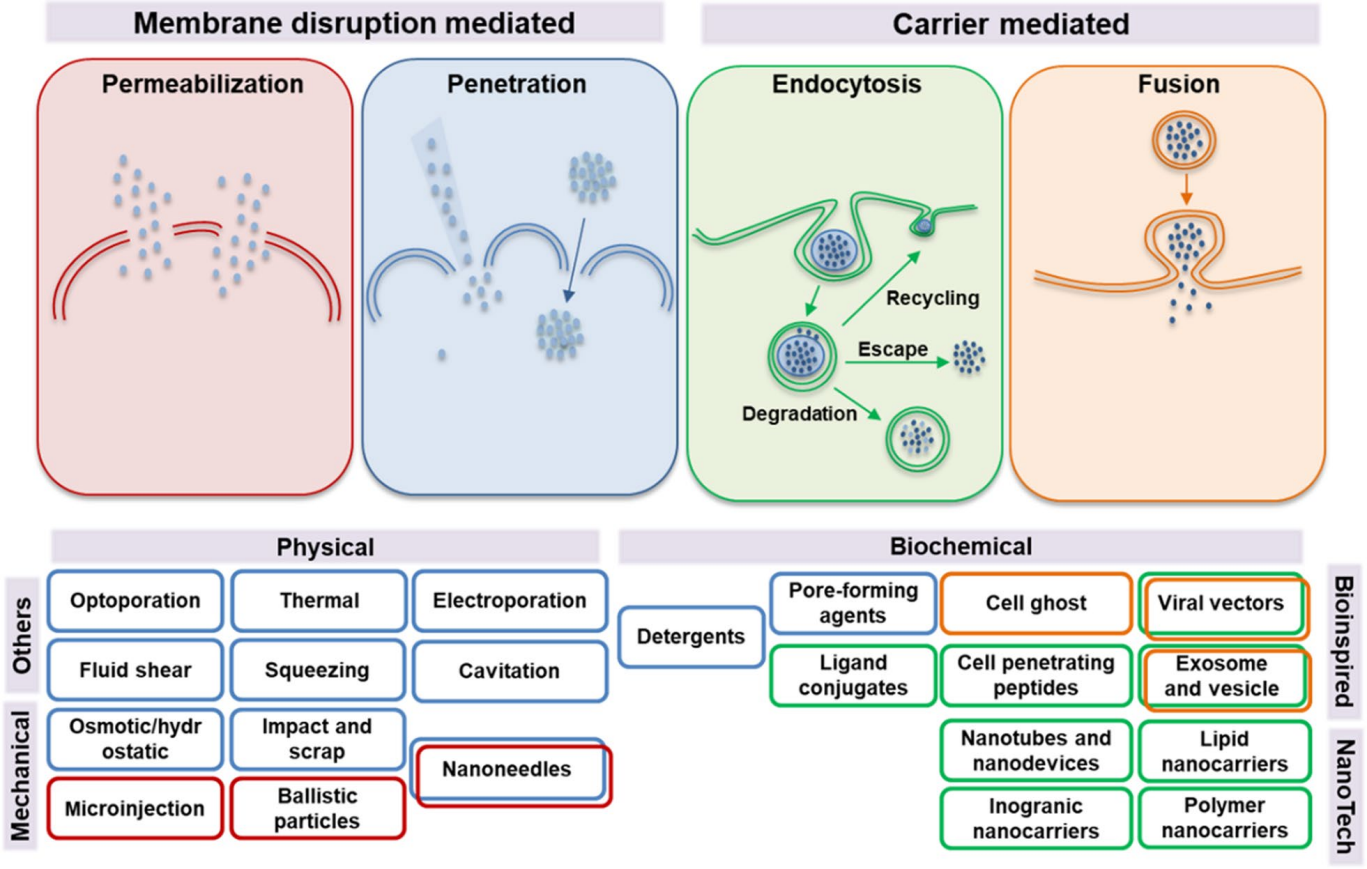
Schematic representation of most commons’ methods for intracellular cargo delivery. Membrane disruption-based methods, such as permeabilization (in red) and penetration (in blue) are physical techniques that induce the formation of transient pores into the plasmatic membrane for the cargo cell internalization. Other methods use biochemical approaches for the membrane permeabilization and the cargo translocation (*e.g.*, detergents, pore-forming proteins). In alternative, cargoes gain the access to the intracellular compartment by carrier-mediated delivery systems (biochemical assemblies or viral vectors). The carrier can be internalized via endocytosis (in green) or by membrane fusion (in orange) depending on its chemical or biological nature

 Intracellular delivery can be achieved by a range of carrier-based or membrane-disruption-based techniques (Fig. 2). Physical and mechanical methods are considered the conventional approach to permeabilize the cell membrane. Microinjection, sonoporation, electroporation, and other techniques have been developed as membrane-disruption modalities to induce transient discontinuities in the plasma membrane using mechanical, electrical, thermal, optical, or chemical forces [26–28]. However, the use of these methods in large-scale treatments and pharmaceutical applications is severely limited by their low-throughput and disruptive techniques that require sophisticated and expensive instrumentations.

As an alternative to mechanical methods, nanocarrier-mediated delivery nanosystems can be designed to respond to the microenvironment changes that trigger the drug release in the target site. Some infection and inflammation-derived conditions, associated with different pathologies, can be exploited as stimuli to promote the disassembly of the nanocarrier, such as variations in pH [29, 30], oxygen [31], or specific biomolecules such as enzymes [32]. In this way, the drug is released specifically inside the target cells or in the target tissue where it acts. The drug release can also be triggered by external physical stimuli such as ultrasounds, light, or magnetic field in a spatiotemporally controlled way [33–36]. Several strategies are being developed in the NP's formulation to combine the biocompatible properties of fully organic nanomaterials with the unique physicochemical properties of inorganic nanomaterials (such as magnetic NPs, plasmonic NP, etc.).

However, once the nanocarriers are taken up by cells via endocytosis, the cargo released from the endosome vesicles is required. Several endosomal escape strategies have been developed based on the endosome rupture promoted by membrane destabilizing agents such as pH-sensitive membrane-perturbing peptides and polymers [37–39]. These molecules are designed to switch their structures and lyse endosomal membranes, under the pH decrease in endosomal compartments during their maturation. The vesicle rupture allows the protein cargo liberation into the cytosol [40]. Natural peptide sequences (for instance, cell-penetrating peptides, CPPs) have been used to facilitate passage through the membrane to specific organelles within the cell. Using a mix of peptides that contain cell permeation or nuclear localization sequences, a wide range of synthetic and biological molecules have been transported into the cytosol [41]. Among the many types of CPPs designed, the arginine-rich CPPs are the most exploited ones [42]. The efficiency of CPP-cargo conjugated is still hard to predict due to the numerous features that could influence it (such as physicochemical properties, local concentration, membrane potential, cell-entry mechanism, etc.) [43]. Recently, a pioneering study introduced a new transport principle based on the chaotropic effect [44]. They utilized globular boron clusters as membrane carriers to transport a broad range of hydrophilic cargo, bypassing the endosomal entrapment.

An alternative solution to achieve intracellular delivery is represented by the direct fusion of a specific nanocarrier with the plasma membrane of cells. This approach is inspired by the natural mechanism viruses use to deliver their genetic material into the cytosol while transfecting their hosts. The mechanisms behind the viral genome release vary among the several family types of viruses. Some envelope viruses, such as herpes simplex virus type I (HSV-I), enter into host cells either by fusion with the plasmatic membrane or intracellularly after internalization via endocytosis [45, 46]. Among the twelve glycoprotein species that compose the membrane envelop of these viruses, five of them (gB, gC, gD, and the complexes of gH and gL) are entry-associated viral glycoproteins and mediate the fusion process between viral and host’s membranes [47]. On the other hand, Haemagglutinin (HA), a coating peptide of the influenza virus, acts as a membrane fusion agent by exploiting the structural modification induced by the typical acidic pH of the lysosomal environment from a hydrophilic and anionic random coil conformation to a hydrophobic helix conformation [48]. This conversion, which typically promotes the fusion of the viral membrane with the cell membrane, can be exploited for endosomal escape strategies.

On this note, virus-inspired systems used for drug delivery have been developed, namely, (i) viral gene vectors, (ii) virus-like particles (VLPs), and (iii) virosomes. Generally, viral vectors are used to deliver therapeutic genes and are mainly applied as engineered vaccines. Retroviruses, adenovirus, adeno-associated virus, herpesvirus, and poxvirus have been selected as gene delivery vehicles thank to their capability to carry and deliver foreign genes [49]. The viral vectors derived from them are employed in more than 70% of clinical gene therapy trials worldwide. Besides the drawbacks linked to the safety of the use of a viral component, these vectors cannot be used as drug carriers due to their limited loading capacity.

VLPs and virosomes are interesting alternatives and more applicable to protein delivery. VLPs are self-assembled capsules that mimic the capsid structure, while virosomes are liposome-like vesicles made of a phospholipid bilayer that is modified to incorporate surface glycoproteins of viruses [50]. Since these systems are not endowed with viral genomes, they are not able to replicate in human cells, which allows overcoming all the safety-related concerns [51]. Recent reports show that VLPs can be used to mediate the efficient delivery of guest proteins to the cytosol [52–54]. In addition, these particles also mimicked the virus ability to overcome biological barriers, including avoidance of opsonization and tumor-homing properties [55]. Chatterjee and co-workers demonstrated effective cytosolic delivery of proteins, such as caspase 8, green fluorescent protein (GFP), and Cre recombinase, using VLPs with group-specific antigen (Gag) fusion proteins on their surface [56]. On the other hand, Savithri and colleagues developed VLPs using *Sesbania mosaic *virus coat protein engineered with Staphylococcus aureus protein A (SpA) to deliver multiple antibodies [57].

### Biointerfacing dilemma

The expectations for translational medical solutions based on nanotechnology are high and the proof-of-concept reports are increasing in the literature. Some first generation of nanomedicines is currently commercialized. These nanoformulations such as Doxil® (pegylated, doxorubicin (DOX)-loaded liposomes, Janssen Biotech, Inc., Horsham, PA), Abraxane® (paclitaxel-containing albumin NPs, Celgene, Summit, NJ), AmBisome (liposomal amphotericin B, Gilead, Foster City, CA), Onivyde®, Marqibo®, and Nanotherm® were approved by the Food and Drug Administration (FDA) and are applied in clinics.[58] The recent SARS-CoV-2 outbreak pushed the development of liposome-based mRNA nanovaccines such as Spikevax ® (Moderna) or COMIRNATY® (Pfizer-BioNTech). mRNA vaccines had been extensively studied and developed in the past few decades [59, 60] and now they have achieved the status of a viable strategy for the treatment of infectious diseases and even cancer [61, 62]. These vaccines represent a revolution in terms of nanomedicine and currently are being explored.

Among nanomaterials, liposomes have shown to be an effective DDS with fewer side effects thanks to their biodegradability, biocompatibility, and easy-to-manipulate size and surface [63]. Liposomes are the DDS that have been more widely approved and commercialized [63, 64]. Their amphiphilic nature allows them to entrap hydrophobic and/or hydrophobic payloads, while high compatibility with the biological environment is allowed by their chemical compositions (phospholipids) and the lipid bilayer structures. However, their instability and aggregation both in blood circulation and in storage have limited their therapeutic applications.

Besides liposomes, a range of other nanomaterial-based formulations is also being studied as potential candidates for vaccine development. For example, self-assembling protein NPs [65], polymeric [66], and inorganic structures [3]. Similarly to liposomes, they offer the possibility for delivery of active molecules and drugs, in combination with other moieties capable of developing specific functions once reached their target sites [67]. Some others non-liposomal NP formulations have reached clinical trials [68] and have been approved for clinical use by the FDA. However, these numbers are still scarce due to the high complexity of the environment that nanoparticles encounter after administration. For instance, as the blood is rich in proteins, they will be adsorbed onto the NP surfaces forming a biomolecular corona. This biomolecular corona gives the NPs a new biological identity, playing an important role in the NP distribution and possibly compromising NPs action [69, 70]. Complement components and immunoglobulins on the NP surface facilitate the recognition and clearance from the bloodstream (opsonization) promoting their fast removal by the organs associated with RES and largely by the macrophage-rich liver limiting their accumulation on the desired site after systemic inoculation. For making NP-based formulations more effective, several issues need to be addressed to improve their long-term stability, degradation, and lack of active targeting, which can limit their application [71, 72]. The surface is mainly responsible for governing interactions with the surrounding environment. For this, increased control in the bio-interface between nanomaterials and biological fluids and barriers must be achieved.

Particles found in nature including cells, viruses, or extracelluar vesicles (EVs) are highly complex and heterogeneous. The biointerfacing capabilities of these natural particles are mainly determined by their membrane layer. The information integrated at their surface has key inherent class properties such as “don’t-eat-me” signals, targeting specific sites, modulating the immune system response, etc. A deeper understanding of cell-cell communication and signaling has led to engineering cell-derived systems as better DDS for wide application in disease treatments, particularly in immunotherapy. For instance, a recent strategy being developed as potential immunotherapy is based on the use of autologous cells modified ex vivo for reinfusion in the patient (adoptive cell therapy), which reduces issues regarding innate immune recognition and clearance. Cell therapies hold great potential for efficacy engineered cellular immunotherapies in cancer. Chimeric antigen receptor (CAR)-T cell therapy, which targets specific cell surface antigen, has been remarkably effective towards certain pathologies such as leukemias and lymphomas [73].

Clearly one of the most important challenges for nanomedicine is to mimic the multicompartmental architecture of cells and the complexity of the cellular membrane. Inspired by this approach, the use of cell-derived membranes in the form of nanomaterials has been proposed. In the last decade, there has been a considerable interest to develop bioinspired nanomaterials derived from biological entities already present in nature. Thanks to their biointerfacing capabilities, cellular structures (i.e., erythrocytes, leukocytes, platelets, and exosomes) or invasive pathogens (i.e., bacteria and viruses) can be exploited to design a new class of nanomaterials to overcome the limitations of synthetic NPs [74]. Among the biomimetic materials, cell membrane-derived NPs offer multiple advantages as drug delivery nanocarriers.

### Biomimetic cell-derived nanocarriers

Recent progress in cell-derived nanosystems has further broadened the nanomedicine tools for advanced therapeutic approaches in imaging, phototherapy, detoxification, immune modulation, and drug delivery [75]. In particular, translating cell membrane features to nanocarriers, such as NPs, liposomes, or other common DDS, offers exciting opportunities to fabricate next-generation of biomimetic nanocarriers for various biomedical applications [76–78].

The complexity and dynamism of the cell membrane go beyond just being a passive lipid bilayer envelope. Cell membrane proteins and carbohydrates (e.g., glycoproteins and glycolipids) are active components of the cellular machinery, and they are the first responders to what surrounds the cell. The cell membrane regulates signaling, transport, and immune response, which has inspired the idea of taking advantage of their intrinsic properties as an integral part of therapies and biomimetic nanoformulations, still exploiting the physical properties related to the synthetic nanomaterial.

Biomimetic nanosystems based on the use of cellular structures have shown numerous advantages compared to synthetic nanomaterials. The use of entities that the body does not recognize as foreign agents, avoids rapid recognition by the RES, leading to a slower clearance and avoiding immune response. Furthermore, there are countless receptor-ligand, binding, and adhesion interactions that can be taken advantage of to achieve high targeting efficiency without applying elaborate functionalization methods. Cell membrane receptor profiles and characteristics are vital in performing therapeutic functions that can be translated onto the membrane coating with no loss in their native functionalities.

On this note, recent studies have been focusing on the possibility to develop DDS using whole cells, cell-derived membranes, or EVs as drug carriers [79].

Whole cell-based nanocarriers have been developed using a different kind of cells such as red blood cells (RBCs) [80], platelets [81], stem cells [82], macrophages [83], or microorganisms [84] to encapsulate drugs, proteins or NPs. Depending on the type of cell source used, specific functionalities can be exploited for the development of tailored systems with different therapeutic functions. Generally, organisms such as bacteria and viruses can be exploited for their infection ability; white cells for the activation of immune pathways; RBCs for their ability to stay in the bloodstream; and tumor cells for homotypic targeting.

However, a limitation in the use of whole cells as nanocarriers is the possibility that drugs loaded on their surface or inside them may be cytotoxic and damage the cell membrane or the whole cell [85], which poses restrictions to the drug dosage to be internalized. To date, cell therapies are very expensive and can also cause clinical syndromes of immunotoxicity and autoimmunity [86], which leads to explore safer, and more affordable immunoregulatory alternative approaches for cell-based personalized medicines. In this direction, the immune system is a highly suitable objective for nanotechnology-based formulations, that have recently attracted the interest of the scientific community and pharmaceutical companies.

In addition to cells, EVs have been recently reported as promising active targeted nanocarriers. These are a heterogeneous group of cell-derived membranous structures comprising exosomes and microvesicles. In particular, exosomes originating from the endosomal system are capable of delivery of various cargos, including proteins, lipids, and nucleic acids acting as communicators that mediate signaling between cells [87]. Thanks to their biocompatibility, cargo protection, long circulation time, and targetability to specific tissue or cells, exosomes have been largely studied as potential DDS of proteins and different RNA species such as siRNA [88] or microRNA [89], drugs [90] and NPs [91]. However, their use has been limited by the lack of standardized methods to rapidly produce, isolate and purify exosomes in sufficient amounts [92].

### Cell membrane sources and their main applications

**Fig. 3:**
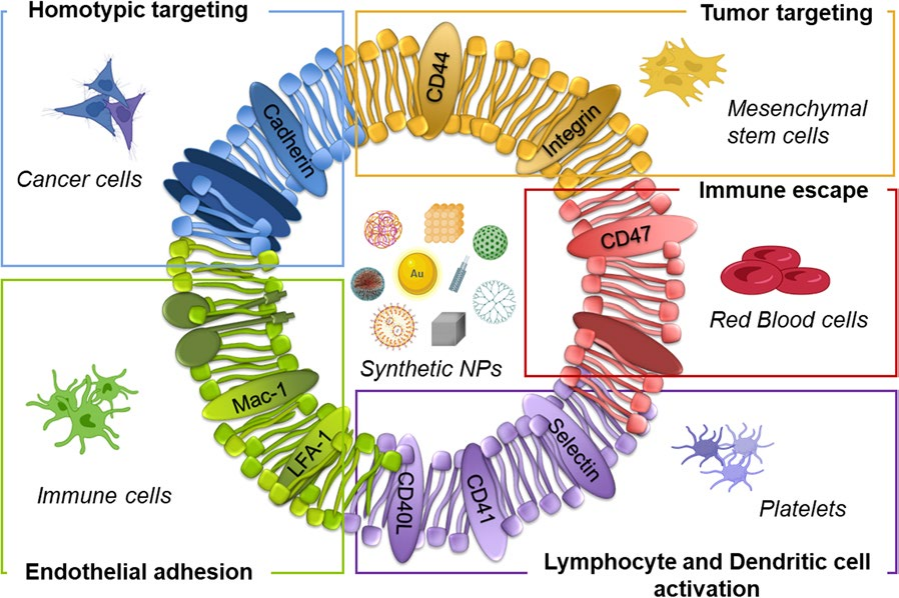
Schematic illustration of biomimetic cell-derived NPs. Depending on the cell type used as membrane coating source, specific features can be exploited for different applications

Most of the targeting and biointerfacing abilities of a cell can be attributed to its plasma membrane. Hence, current efforts are focused on the development of core-shell structures by using plasma membrane of different cell sources as biomimetic coating for synthetic NPs (see Table 1). Cellular membranes used for NP camouflaging are generally isolated from blood cells, immune cells, cancer cells, and stem cells. The presence of specific moieties involved in recognition, adhesion and interaction mechanisms makes these different cell types suitable for several applications in the field of tumor theranostics (Fig. 3).

#### Red blood cells

Thanks to their interesting properties, such as prolonged blood circulation time, absence of nuclei, and abundance in the body, RBCs represent the more exploited source of cell membrane coatings. Zang and co-workers were pioneers to explore RBC membrane (RBCM) camouflaged NPs for cancer therapy. In their first study, biomimetic NPs composed of poly(lactic-*co*-glycolic acid) (PLGA) NPs combined with RBCM purified from fresh RBCs showed a half-life significantly longer than PEGylated-PLGA (39.6 vs. 15.8 h) with an in-blood retainment even 72 h long after injection [93]. Improved pharmacokinetic behavior of RBCM-camouflaged NPs is mainly due to the expression of specific membrane proteins, such as CD47 that inhibit the phagocytosis by macrophages residing in the RES system (liver, spleen, and lungs). CD47 is also known as a *don’t eat me* signal and is responsible for the RBCM ability to escape the recognition by the immune system and minimize premature blood clearance, phenomena observed in PEG functionalized NPs [94]. Besides the biological advantages, the RBCM coated-NPs have shown to improve structural rigidity and particle stability, also leading to a more reliable cargo encapsulation [95, 96].

RBCMs have recently been used for cancer immunotherapy, leveraging host anti-cancer immune reactions [97] Liang et al [98]. developed biomimetic-based photothermal cancer immunotherapy using a nanoformulation of RBCM-derived black phosphorus (BP) quantum dots (QDs) nanovesicles (BPQD-RMNVs) combined with Programmed Death-1 antibody (aPD-1); they induced apoptosis in cells, in situ by near-infrared (NIR) laser irradiation and neoantigen release-mediated immune system activation to eliminate residual and metastatic cancer cells. The NIR mediated apoptosis promoted the recruitment of dendritic cells (DC) and the neoantigens release. Subsequently, an intensive tumor-specific CD8^+^ T cells response was activated against primary and secondary tumor growth. BP-mediated photothermal therapy (PTT) combined with checkpoint antibody treatment has promise as a potential clinical treatment for breast cancer [98].

#### Platelets

Another type of cell membrane that can be extremely useful in biomimetic nanotechnology development is the platelets-derived one. The platelets originate in the bone marrow and are involved in numerous vital process as homeostasis, tissue repair, and thrombosis as well as inflammation and adaptive and innate immune responses [99, 100] Platelets have a crucial role in cardiovascular disease and carcinogenesis too [101, 102]. In fact, platelets membrane (PM) expresses proteins such as D-selectin that recognize and interact with CD44-overexpressing circulating tumor cells (CTCs), which are strictly involved with tumor metastasis and angiogenesis [103]. Hu et al. developed PM-coated core-shell nanovesicles (PM-NVs) loaded with (i) tumor necrosis factor (TNF)-related apoptosis factor (TRAIL) and (ii) DOX. In this formulation, the TRAIL was efficiently delivered toward cancer cell membrane where it activated the extrinsic apoptosis signaling pathway.

**Table 1:** Currently explored source cells for membrane-coated NPs

Source	Role	Main Surface Markers	Coated Nanocarrier
RBCs	Tumor targeting; removal of pathogens; long-term circulation.	CD47;	PLGA; [93] [94, 104] QDs; [98] Polymeric NPs; [105] Mesoporous Silica NPs; [106] Gold NPs (AuNPs); [107]
Platelets	Immune escaping; Atherosclerotic site targeting; Injured vessels and tumor targeting.	CD47; Membrane glycoproteins; Selectin.	Nanogels; [108] [109] QDs; [110] Magnetic NPs; [111] PLGA; [112]
Neutrophils	Extravasation from blood vessels; Inflammation and infection targeting; Tumor targeting.	L-Selectin; P-Selectin; Macrophage antigen-1; LFA-1; VLA-4.	Liposome; [113] PLGA; [114]
Lymphocytes	Extravasation from blood vessels; Immune system evasion; Tumor targeting.	LFA-1; CD11a.	Nonporous silicon particles; [115] (leukolike) Lipidic NPs; [116]
Macrophages	Antitumor properties.	CD86; CD80; MHC-II.	Nanoporous silicon particles; [117] Gold Nanoshells (AuNSs); [118] Upconverting NPs (UCNPs); [119]
Mesenchymal cells	Inflamed site targeting; Tumor targeting.	CD44; Integrins.	Nanoporous silicon particles; [120] Nanogel; [121]; Polymeric NPs; [122]
Cancer cells	Homotypic tumor targeting; Immune system evasion; Anti-cancer vaccines.	Galectin-3; Cadherins; Integrins; CD326 (EpCAM); TF-antigen; CD44.	Magnetic Iron oxide NPs; [123] PLGA; [124], [125] UCNPs; [126] Mesoporous silica NPs; [127] AuNPs; [128]
Bacteria	Stimulating innate immunity Promoting adaptive immunity Tumor targeting	Immunogenic antigens; Pathogen associated-molecular patterns (PAMPs).	AuNPs; 129] PLGA; [130] FeO; [131] Polymeric NPs; [132]

Simultaneously, equipped with an acid-responsive encapsulation matrix, PM-NVs can be digested after endocytosis and enhanced the DOX accumulation at the nuclei for activation of the intrinsic apoptosis pathway [108].

The same group, to improve the drug accumulation in tumors, developed a new strategy combining two nanocarriers. For the first one, they used Arg-Gly-Asp (RGD) peptide to decorate tumor necrosis factor α (TNF-α) loaded nanovesicles. The RGD peptide selectively binds the integrins such as *ανβ*3, overexpressed in tumor blood vessels, while TNF-α, an inflammation-induced cytokine, is applied to trigger tumor vascular damage. The second nanocarrier is a PM-coated acid-responsive dextran nanostructure loaded with the chemotherapeutic agent Paclitaxel (PTX). The study showed that PM proteins such as CD36, CD42d, P-Selectin, and CD40L were efficiently transferred with their origin membranes and enriched the NP coating. Thanks to the specific interaction between the L-Selectin and the CD44 receptor of the tumoral cells, the authors demonstrated that the presence of the PM gives to the NPs the ability to target the myeloma cells with high internalization, high intracellular drug localization, and decreased side effect [109].

#### White blood cells

Biomimetic coating for NPs has recently also been obtained from membranes extracted from white blood cells (WBCs), which are recruited into the tumor site in relation to chronic inflammation. First studies showed that this approach can enhance immune evasion and inflammation targeting [115]. ‘Leukolike vectors’ (LLVs) retain many critical leukocyte transmembrane proteins from the cell donor [117], which by clustering reduce RES uptake; others are involved in the adhesion to inflamed endothelium and tumor targeting, or in immune tolerance and interaction with platelets [133, 134].

Thanks to the presence of lymphocyte function-associated antigen 1 (LFA1 or CD11a), coated NPs bind actively the TNFα-activated endothelium as evidenced by clustering of endothelial intracellular adhesion molecule 1 (ICAM-1). Furthermore, a transwell chamber assay showed the high suitability of LLVs to cross the layer of inflamed endothelium [115].

Leukocyte membrane coating was also used for designing nanoformulations for imaging and PTT. Xuan et al. [118] successfully developed macrophages membrane coated (MPCMs) AuNSs for PTT cancer therapy. In mice, the macrophagic coating demonstrated a significant biocompatibility increase, opsonization reduction, circulating time prolongation, and tumor-tropic accumulation of MPCM-AuNSs enhancement. Moreover, in vivo PTT showed the inhibition of tumor growth upon NIR irradiation and even its disappearance after 25 days.

#### Mesenchymal cells

Mesenchymal cells (MSCs) are multipotent stem cells with the ability to differentiate into other types of cells such as adipocytes, fibroblasts, osteoblasts, chondroblasts, and pericytes. Furthermore, the MSCs migrate to the injured and inflamed tissue under environmental conditions such as hypoxia, and interaction with Toll-like receptors or cytokines. Since the tumor is considered to be a chronic inflammation disease, MSCs membrane coated NPs strategy has been harnessed as a biomimetic approach for targeted delivery of cancer drugs to tumors [120, 135]. Toledano Furman et al. [136] developed MSCs membrane-derived carriers as model platforms entrapping therapeutics and achieving specific tumor targeting and tumor growth inhibition. On the other hand, synthetic liposomes as negative control did not show analogous results. It was suggested that the carriers´ ability to bind and fuse to the tumor cell surface was due to the presence of specific MSC integrins, retained on the membrane coating that can mediate cell-derived nanocarriers interaction with the tumor-infiltrating immune cells, blood vessel endothelium, and tumor-associated fibroblasts [136]. Similarly, Changyong and co-workers used MSCs membrane to coat a gelatin nanogel loaded with DOX. Their studies demonstrated high tumor-targeting capability both in vitro and in vivo of their nanosystem. The MSCs membrane coating significantly improved the cellular uptake, intratumoral accumulation, and penetration compared with gelatin-DOX and free-DOX administration [121].

#### Cancer cells

Besides RBCs, platelets, WBCs, and MSCs, cancer cells present exciting advantages to be exploited as membrane coating against tumors. Camouflaging strategies based on their use take advantage of innate homotypic aggregation properties and the immune escape ability.

Homotypic targeting is the intrinsic ability of cancer cell coating nanoformulations to interact preferentially and strongly with the same cells from which they are originated. This feature provides a unique asset for any specific targeted drug delivery strategies against cancer. The homotypic affinity between cancer cells can be attributed to the interaction between galectin-3 and carcinoembryonic antigen expressed on cancer cells [137]. Fang et al. [124] first studied the homotypic targeting of MDA-MB-435 cancer membrane-coated PLGA NPs as DDS. The coated-NPs showed a 20-fold increase accumulation in MDA-MB-435 cells compared with the bare PLGA NPs, while no difference was observed in human foreskin fibroblasts. Therefore, an analogous formulation was performed using a non-specific RBC coating PLGA NPs and it showed reduced particle binding to the cancer cells, suggesting that the cancer cell coating enhanced particle-cell adhesion [124].

A similar approach was applied by Sun and colleagues developing a 4T1 cell membrane-coated paclitaxel-loaded polymeric core as biomimetic (CPPNs) DDS against breast cancer and its metastasis in the lungs [125]. 4T1 coated CPPNs preferentially targeted its tumoral cells but not lung fibroblast WML2 cells or macrophage RAW264.7 cells. The accumulation of that nanoformulation in the primary tumor and metastasized site in the lungs increased by 3.3- and 2.5-fold when delivered with CPPNs instead of bare NPs. Furthermore, RBC-coated PPNs (RPPNs) and synthetic liposome vesicle-coated PPNs (LPPNs) were used as a negative control, and they exhibited a rather lower uptake than the CPPNs, suggesting that the enhanced internalization of the CPPNs was probably caused by the 4T1-tumor-cell-membrane proteins obtained from the source cells. The membrane proteins including TF-antigen and E-cadherin associated with the adherence capabilities during the colonization of metastasis lesions also have effects on the homotypic interactions among the tumor cells [138–141]. CD44 and CD326, the surface adhesion molecules on the 4T1 cells, have been recognized as surface markers that also played main roles in the adherence of the metastatic cells to the distant sites [142, 143].

Many other results regarding the exciting specific interaction capabilities leveraged by cancer cell coating have recently been reported in the literature [126, 128, 144] Besides cancer-targeted drug delivery, cell membrane-coated NPs could also be exploited for developing novel bio-synthetic nanocarriers for vaccines. Because many tumor antigens are surface markers, the tumoral cell membrane can activate the immune system to recognize and kill malignant tumor cells based on variant antigen expression [145, 146]. Approaches based on a single tumor-associated antigen can be inadequate when facing the high heterogeneity and mutation rate of cancer cells [147]. On the other hand, when cell lysates are used in multiantigen-based strategies to prime the immune system, the large presence of intracellular, housekeeping proteins may divert focus away from the relevant antigens, which compose a small percentage of the total protein, thus compromising the treatment efficacy [148]. Therefore, cancer cell-coated NPs represent a good approach to combine the homotypic capability to recognize the tumoral cells and the active delivery of tumor-associated antigens to DCs for immune processing, which allowed for the subsequent stimulation of tumor antigen-specific T-cells [124].

#### Bacteria

Besides mammalian cells, also pathogens cells can be a source of membranes for NP coating. Many studies have drawn attention to bacteria as a novel delivery system for various biomedical applications [149]. While many approaches for the design of NP-based vaccines are based on including immunostimulatory ligands, bacterial membranes are by nature immunogenic. They contain potent pattern recognition receptor (PRR) ligands that play a key role in stimulating innate immunity and promoting adaptive immune responses [150, 151]. Coating the NPs with such molecular patterns will transfer their intrinsic adjuvant properties [152]. In consequence, professional antigen-presenting cells (APCs) will process them in a similar way to the source pathogen cell, along with the antigens co-administered.

Zhang’s group applied *E. coli* as a model pathogen to coat AuNPs and produce an antibacterial vaccine [129]. Specifically, they used bacterial outer membrane vesicles (OMVs), also known as extracellular vesicles. OMVs are naturally produced from all Gram-negative bacteria and have nano-sized lipid-bilayered vesicular structures composed of various immunostimulatory components [153, 154]. They showed stronger activation of CD11c^+^ DCs cells of the bacterial membrane-coated AuNPs compared to the OMVs. In addition, both humoral (antibody titers) and cellular (levels of interferon-γ IFNγ and interleukin 17 IL-17) responses in vivo against *E. coli* were more pronounced. These findings were followed by other research lines aimed to use OMVs as immunotherapeutic agents against tumor.

In 2017, Kim et al. reported that OMVs from *E. coli* accumulate in tumor tissues in vivo, and elicit strong activation of IFNγ-mediated long-term anti-tumor immune response [155]. Recently, Cheng et al. developed engineered OMVs co-expressing specific tumoral antigens and promoting the maturation of DCs to trigger a subsequent antigen-specific T-cell-mediated adaptive immune response [156]. They showed that by functionalizing OMVs with tumor antigens through genetic engineering they can develop tumor-targeted vaccines. Such engineered OMVs could control B16 melanoma lung metastasis and inhibit subcutaneous colorectal cancer growth by sustaining and efficient antigen delivery to the lymph nodes, with the subsequent maturation of DCs. The OMVs already constitute nanosized vectors with inherent adjuvant functionality. Therefore, OMVs transfer and functionalization to other kinds of NPs containing other drugs/antigens could render great multifunctional vectors.

Despite the growing number of research aimed at developing these new biomimetic systems, the mechanisms involved in biointerfacing with the biological environment, i.e., how these membrane coatings interact with cells at the molecular level, still require in-depth studies. In addition, because in most cases the cargo consists of drugs that need to reach the cytosolic target, it is crucial to investigate the intracellular delivery mechanisms.

### Future perspective and conclusion

Trafficking processes, access to barriers and into specific cellular locations is governed by active biological recognition. Despite synthetic nanocarriers can be designed with variable features such as size, shape, surface charge and functional molecules properties, and responsiveness to deliver a specific therapeutic; a very significant element of the limitations experience so far in actively targeted nanomedicines, derives from the fact that insufficient tools have been available to address the complex role of biological interactions.

Natural nanostructures are highly structurally and functionally heterogeneous and complex. They present key inherent class properties to develop precision nanomedicines. For instance, the complexity and dynamism of the cellular membrane can be translated to nanocarriers offering the capability of overcoming heterogeneous biological barriers. Such biomimetic interfaces have emerged to overcome the main drawbacks inherent to synthetic nanomaterials. Cell membrane-derived nanostructures hold great promise as bio-inspired synthetic nanocarriers for vaccines and drug-delivery systems. These cell-derived nanocarriers can offer a powerful toolbox for cancer treatment, combining their intrinsic properties with existing therapeutic strategies, such as phototherapy, immunotherapy, gene therapy, and chemotherapy [157]. Cancer causes uncontrolled growth of cells in the different organs and tissues of the body. Moreover, CTCs can be spread to the blood and cause metastasis. Personalized cancer treatment for individual patients is among the main aims of future therapies. Multiple types of cells can be exploited to provide versatile biomimetic nanoplatforms for advanced personalized therapeutic agents. A variety of immune-associated cells (natural killer cells, macrophages, DCs, etc.) can be found in the tumor microenvironment. Recent studies have also demonstrated the role of bacteria in the tumor microbe microenvironment [158, 159]. Therefore, selecting the right cell source (RBCs, immune cells, cancer cells, bacteria, etc.) allows for mimicry of their inherent natural properties and native functionalities as we described above. The cancer cell membrane is known for its homotypic targeting abilities due to the adhesion molecules present at the surface. Engineering cancer cell-derived nanocarriers could deliver chemo drugs to specific tumors or metastatic sites avoiding unwanted side effects. Additionally, cell surface modification by metabolic or gene engineering and lipid insertion enables the introduction of other functionalities such as incorporating neoantigens or other active targeting moieties or chemo drugs. Hybrid-cell-derived carriers by fusion of different cell membranes allow merging biological properties of different cells [160]. For instance, incorporating the properties of RBC membrane fragments into cancer cell membranes will enhance circulation time [161], or platelet cell membranes will help to specific target CTCs [112].

These tunable biomimetic properties combined with adequate organic or inorganic nanomaterials could offer new synergistic benefits of cell-based delivery nanosystems in the fields of bioimaging and therapy with a special focus on cancer treatment.

For instance, coating inorganic NPs with the right biomimetic nanocarriers can be exploited for photodynamic therapy (PDT) and PTT in cancer treatment. PTT can be used to kill cancer cells utilizing light energy of the NIR wavelength range to generate localized heat. Combining cell-derived carriers with the tunable features of AuNPs, which also have strong interactions with light, make them efficient alternatives for use in PTT [128]. PDT uses a drug (a photosensitizer) activated by light to generate reactive oxygen species (ROS) and kill cancer cells. Importantly, multifunctional carriers based on UCNPs, silica NPs or metal-organic frameworks (MOFs), in combination with photosensitizers such as Chlorin e6 or porphyrins have been camouflaged by cell membrane coating for PDT applications [162]. Cell membrane coatings allow for lowering the dose of the inorganic nanomaterials and drugs contributing to decrease toxicity and minimizing side effects to the surrounding healthy tissues.

Immunotherapies have a huge potential for clinical success in cancer treatment. The use of immune cell-derived nanocarriers will allow for enhancing immune responses by delivering immunomodulators and activating T cells or by delivering adjuvants and tumor antigens to DCs for tumor vaccination. Recent research has been focused on delivering tumor neoantigens with the aim to activate the patient’s immune system to recognize and kill tumor cells (tumor vaccination) [163].

Biomimetic nanocarriers have the potential to improve cell targeting and precision delivery of drugs in vivo, can be surface engineered, and have a large capacity for cargo encapsulation and stabilization. It has been demonstrated that cell membrane coating of nanocarriers reduces immune activation, increases blood circulating time, and shows tumor-homing capacity. Most of the studies reported their in vivo application in mouse models where they had shown good biocompatibility and safety. However successful clinical applications require understanding how they behave at the cellular level. Fundamental investigations of biomimetic nanocarriers and the resulting interactions within the tissues and cells are not completely elucidated yet and need to be more thoroughly and systematically analyzed. On one hand, identifying critical motifs responsible for targeting surface markers and cell receptors is essential for the rational design of efficient nanocarriers to target specific tissues. On the other hand, a better understanding of the cell internalization mechanisms opens new opportunities for intracellular applications that require an endosomal escape. Investigate and predicting these bio-molecular mechanisms require the implementation of more sophisticated and reliable three-dimensional cell culture models (spheroids and organoids cultured under flow conditions, etc.) closer to natural conditions. Recreating a 3D microenvironment as cell-cell and cell-extracellular matrix interactions offers a bridge between in vitro 2D cell culture and animal testing before moving to clinical translation. These new platforms will allow understanding the complexities of in vivo cellular behavior of cell-derived nanocarriers. As it is described along this review cell membrane-coated nanocarriers have immense potential to enhance drug delivery mechanisms however critical limitations need to be overcome before being translated to clinical trial stages (see Table 2). Large-scale production and standardization protocols for manufacturing and characterization processes of these biomaterials are one of the key challenges. For instance, different types of cells require different cell culture conditions. The quantity and quality of the sample preparation could depend on cell type, cell cycle, cell lifetime, passaging, etc. Not only reducing batch-to-batch reproducibility is important, but biomimetic NPs may also be obtained in broad populations. Due to the heterogeneity of the cell surface, nanocarriers with different motifs or densities of specific proteins or motifs may be synthesized in the same batch. Therefore, there is a requirement for established well-defined characterization tools for analyzing the surface composition and biological efficacy. It is also essential to ensure the cell surface integrity and the presence and correct orientation of key membrane proteins and long-term stability, after the assembly of the cell membrane fragments. The targeting ability may be impaired due to the loss of proteins or their functionality during cell membrane extraction and purification or storage.

Cell-derived nanocarriers are suitable candidates for cancer therapy but it also offers the versatility to develop personalized nanomedicines. A variety of cellular sources can be exploited to design a library of biologically derived membranes with unique properties. They also can be loaded with a combination of cargoes, either several drugs or a combination of drugs and inorganic NPs. However, the use of natural components continues to raise safety concerns such as potential immunogenicity. Overall, we expect this emerging cell membrane-coated technology capable of mimicking natural scenarios will open a new class of nanocarriers for targeted cancer therapy and other biomedical applications.

**Table 2:** Advantages and limitations of biomimetic cell-derived nanocarriers

Advantages of biomimetic cell-derived nanocarriers	Limitations of biomimetic cell-derived nanocarriers
Biological compatibility Stealth properties (reduce non-specific interactions) Tuneable physical, chemical and biological properties (different cell sources, inherent biological properties) Biointerfacing capabilities. Ability to retain cellular properties Improve accumulation and efficacy at the target site (like solid tumors) Immune escape (evading uptake or clearance by the reticuloendothelial system) Prolonged circulation time Protect the encapsulated cargo	Large scale-up (sterile conditions, reproducibility) Standardized protocols (for cell culture to specific cell type) and well-stablished characterization tools Safety concerns (immunogenicity and virulence) when they are derived from pathogens Long term storage Heterogeneity (methods to validate their structural integrity and functionality) Quality control (contamination: microorganisms, denatured proteins)

## Data Availability

Not applicable.

## Abbreviations

APC: Antigen presenting cell
aPD-1: Programmed death-1 antibody
AuNS: Gold nanoshell
AuNPs: Gold nanoparticle
BP: Black phosphorus
BPQD-RMNVs: RBCM-derived black phosphorus QDs nanovesicles
CAR: Chimeric antigen receptor
CPP: Cell-penetrating peptides
CPPN: Cancer cell membrane-coated paclitaxel-loaded polymeric core nanoparticle
CTCs: Circulating tumor cells
DC: Dendritic cells
DDS: Drug delivery systems
DOX: Doxorubicin
EGF: Epidermal growth factor
EPR: Enhanced permeability and retention effect
EV: Extracellular vesicles
FeO: Iron oxide nanoparticles
FDA: Food and Drug Administration
Gag: Group-specific antigen
GFP: Green florescent protein
HA: Hemagglutinin
HIV-1: Human immunodeficiency virus type 1
HSV-1: Herpes simplex virus type I
ICAM-1: Endothelial intracellular adhesion molecule 1
IFNγ: Interferon-γ
IL-17: Interleukin 17
LDA: Laser doppler anemometry
LFA-1: Lymphocyte function-associated antigen 1
LLV: Leukolike vector
LPPN: Liposome vesicle coated paclitaxel-loaded polymeric core NP
MHC-II: Major histocompatibility complex II
MOFs: Metal-organic frameworks
MPCM: Macrophages membrane coated nanoparticle
MSC: Mesenchymal cell
NIR: Near infrared
NP: Nanoparticle
OMVs: Outer membrane vesicles
PAMPs: Pathogen associated-molecular patterns
PDT: Photodynamic therapy
PEG: Poly(ethylene glycol)
PLGA: Poly(lactic-co-glycolic acid)
PM: Platelet membrane
PM-NV: Platelet membrane-coated core-shell nanovesicles
PRR: Pathogen recognition receptor
PTT: Photothermal therapy
PTX: Paclitaxel
QDs: Quantum dots
RBC: Red blood cell
RBCM: Red blood cell membrane
RBCNP: Red blood cell coating PLGA nanoparticle
RES: Reticuloendothelial system
RGD: Arg-gly-asp peptide
ROS: Reactive oxygen species
RPPN: Red blood cell-coated paclitaxel-loaded polymeric core nanoparticle
SpA: *Staphylococcus aureus* protein A
TNF: Tumor necrosis factor
TNF-α: Tumor necrosis factor α
TRAIL: Tumor necrosis factor-related apoptosis factor
UCNP: Upconverting nanoparticle
VLP: Virus-like particles
WBC: White blood cell
